# Jeopardies of Aversive Leadership: A Conservation of Resources Theory Approach

**DOI:** 10.3389/fpsyg.2018.01935

**Published:** 2018-10-17

**Authors:** Tasneem Fatima, Mehwish Majeed, Syed Z. A. Shah

**Affiliations:** International Islamic University, Islamabad, Islamabad, Pakistan

**Keywords:** aversive leadership, work alienation, job performance, psychological capital, conservation of resources theory

## Abstract

The research on the dark side of leadership is still in its infancy. We have contributed to this line of research by proposing that work alienation acts as an underlying mechanism through which aversive leadership results in reduced job performance. We further propose that psychological capital (PsyCap) acts as an important personal resource that reduces the negative effects of aversive leadership in the form of work alienation. The proposed model gets its support from the conversation of resources theory given by Hobfoll (1989) which suggests that stressful situation like an aversive leadership results in the loss of employee resources as a result of that he/she indulges in work alienation and shows poor job performance to retain back the lost resources. People with better personal resources in the form of PsyCap are better able to cope-up with the aversive leader behavior and make them able to avoid work alienation. It is a time-lagged study. The data for the current study was collected from 321 employees working in the service sector organizations, particularly universities, banks and telecom organizations, through personally administered questionnaires. The results supported the mediation and moderation hypothesis. Limitations and future research along with theoretical and practical implications are given at the end.

## Introduction

Leadership, as a universal phenomenon, has gained much attention from researchers as well as practitioners who have come up with different approaches to study this concept from time to time (Antonakis, 2017). The early provokers of the concept talked about the romance of leadership to explain it as a positive force (Bligh et al., 2007). However, with an increase in the lawsuits and ethical misconduct on the part of leaders, researchers are starting to realize that a leader may not always possess “heroic” abilities (Pearce et al., 2008). This has led to the development of research interest in the dark side of leadership. The supporters of this recent trend believe that sometimes a leader may act as destructive and dysfunctional not only for the subordinates but also for the organizations (Skogstad et al., 2017; Barelds et al., 2018; Nevicka et al., 2018).

A careful analysis of the existing literature reveals that with only a few exceptions (Pearce and Sims, 2002; Bligh et al., 2007; Spain et al., 2016; Skogstad et al., 2017), leadership research is saturated with studies highlighting the positive side of a leader. However, with an increase in awareness regarding the costs associated with dark leadership style and its negative and dysfunctional consequences, researchers are realizing that there is a strong need to examine dark leadership styles in different contexts (Karakitapoğlu-Aygün and Gumusluoglu, 2013; Arnulf et al., 2016; Schmid et al., 2018).

According to a recent meta-analysis conducted on destructive leadership, there is a dire need for further research to examine the underlying mechanism how and why destructive leadership styles become detrimental for subordinates (Schyns and Schilling, 2013). It was also identified in the study that very few researchers examined the impact of dark leadership on employee performance that urges to study the subordinates’ poor performance due to destructive leaders. We contribute to this line of literature by studying an underlying mechanism - the work alienation between dark leadership style that is aversive leadership and job performance. This mechanism has been theorized on the conservation of resources (COR) perspective (Hobfoll, 1989) which explains how people with better psychological resources can better deal with the dark leader. The aversive leader is the one who abuses his/her leadership power and frequently uses tactics such as intimidation, threats, and reprimands while dealing with his employees (Pearce et al., 2003; Bligh et al., 2007; Thoroughgood et al., 2011). We propose that aversive leadership leads to a decrease in job performance and work alienation mediates this relationship.

The research on dark leadership styles is in its initial stages and most of the studies on this line have taken into account factors such as emotional exhaustion, job stress, organizational deviance, and other withdrawal behaviors (Skogstad et al., 2007; Wei and Si, 2013; Nauman et al., 2018) while ignoring an important aspect of withdrawal state that is work alienation. Although work alienation has been linked with positive leadership styles such as supportive leadership (Banai and Reisel, 2007) transactional and transformational leadership (Sarros et al., 2002), the existing literature is still silent on its relationship with dark leadership styles. This literature gap rationalizes to examine work alienation as a mediator. Moreover, there is a dire need to examine new and unique underlying mechanisms through which aversive leadership leads to different work outcomes particularly in developing economies (Saeed et al., 2017).

Work alienation is defined as the difference between the employee perception regarding work activities along certain dimensions (power, meaning, and self-expression) and their expectations with regard to these dimensions (Mottaz, 1981). Work alienation has not received the importance it deserved from the academicians. It is urged to conduct research on the impact of leadership on work alienation (DiPietro and Pizam, 2008). Similarly, a meta-analysis on work alienation also suggested the researchers to extend research on work alienation (Chiaburu et al., 2014). Many researchers have highlighted a need to study its antecedents and consequences (Banai and Reisel, 2007; Nair and Vohra, 2010; Shantz et al., 2015; Fedi et al., 2016). In addition to this, the COR theory also supports the notion that stressful work events lead to loss of resources (Hobfoll, 1989) in the form of work alienation comprising of meaninglessness at work, powerlessness, and self-estrangement. Keeping this in view, the current study has taken work alienation as an intervening mechanism through which aversive leadership leads to a decrease in employee job performance.

Before going into the further details about the aversive leadership and its consequences, it is very important to differentiate it from other destructive leadership styles. The research stream on dark leadership styles has identified several negative leadership behaviors in addition to aversive leadership. They include but not limited to destructive leadership (Einarsen et al., 2007; Schyns and Hansbrough, 2010), abusive supervision (Tepper, 2000), narcissistic leadership (Brunell et al., 2008), supervisor undermining (Duffy et al., 2002), despotic leadership (Aronson, 2001), and petty tyranny (Ashforth, 1994; Wong et al., 2017). Although all dark styles of leadership shares a common theme but vary from each other in terms of target, the perception of followers, intent of the leader, frequency, and intensity of the negative behavior, and verbal and non-verbal (Schyns and Schilling, 2013). This meta-analysis could report only a single study on aversive leadership. Aversive leadership is based on punishment orientation and admonishments, both characteristics differentiate it from other dark sides of leadership such as despotic, directive, autocratic, and unethical leaders who are more indulged in the exercise of position power and attainment of their selfish interests/objectives.

Although aversive leadership shares a few of its characteristics with abusive leadership both of which target employees, are measured in terms of perception of employees and display verbal and non-verbal behavior. But the important difference between the two interrelated but distinct concepts is that abusive supervision is an umbrella term that includes a wide range of verbal and non-verbal behaviors such as public ridicule, silent treatment, and blame game as suggested by Tepper (2000) whereas aversive leaders only rely on a narrow list of actions to get what they want such as threats, punishment, and reprimands (Bligh et al., 2007).

Moreover, few negative leadership styles are focused on subordinates and organization, for example, despotic leadership and tyrannical leadership whereas aversive leadership is focused on subordinates only. Additionally, aversive leaders are not manipulative such as despotic, machiavellian, and narcissist leaders. These few characteristics distinguish it from other dark leadership styles.

The scarcity of research on dark leadership style particularly aversive leadership calls for more research to understand this important yet understudied phenomenon (Schyns and Schilling, 2013). With support from COR theory (Hobfoll, 1989), we are responding to the repeated call for research on aversive leadership and work alienation by linking aversive leadership to job performance *via* work alienation. We have also proposed that personality dispositions affect the way employees react to aversive leadership. Specifically, we suggest that employees with high psychological capital (PsyCap) are less likely to experience work alienation under an aversive leader due to their personal resources.

### Conservation of Resources (COR) Theory as Theoretical Foundation

Past studies have used many theories to explain dark leadership styles with negative work outcomes, for example, transactional theory of stress by Lazarus and Folkman (1984) which defines stress as an imbalance between demands and resources and explains stressor appraisal process, cognitive categorization theory by Crocker et al. (1984) which states that individuals develop different categories based on their experience of the world and this affects their behavior, and Barbuto (2000) theory of follower compliance which talks about the psychological processes that motivate employees to comply with a destructive leader and so on (Shaw et al., 2011; Thoroughgood et al., 2012). However, we have used the COR theory (Hobfoll, 1989) for supporting our hypotheses. The major difference between COR and other theories is that COR theory talks about the potential or actual loss and gain of resources whereas other theories treat stress differently. The reason behind taking COR theory as a supporting mechanism instead of other theories is that our proposed model talks about the loss and accumulations of resources. Work alienation indicates the loss of resources due to aversive leader and employees are not able to better perform in their jobs. We have proposed PsyCap as a personal resource that helps to avoid resource losses and help to avoid withdrawal behaviors. In line with these resource loss, accumulation, and retention mechanisms, we believe that COR theory justifies our proposed model better as compared to the other theories.

The COR theory suggests that individuals continuously strive to “seek, acquire, and maintain” resources. This framework explains that people react to the situation in which there is the threat of a loss of resources, an actual loss in resources, or lack of an expected gain in resources where resources can be “objects,” “energies,” and personal characteristics.” The reaction mostly manifest into withdrawal states until and unless they gain some resources to cope up with the resource losses.

Employing COR theory, we argue that aversive leadership is a stressful situation the subordinates encounter with and they feel the loss of resources in the form of their alienation from their work. More specifically due to threatening and punishing behavior of leader’s behavior, they feel no control over their own behavior and indulge into a state of powerlessness. When they are threatened to perform certain tasks, they don’t feel any meaningfulness in their jobs and they get disengaged and avoid self-expression. All these states indicate work alienation that further hinders them to perform better due to resource losses.

Personality dispositions or individual differences are considered an important component of the COR mechanism. COR theory suggests that individual differences can be taken as personal resources (Hobfoll, 1989, 2001). These resources vary from one person to the other. This difference in the level of these resources determines how different employees react to stressful situations. Relying on COR theory, we propose that PsyCap acts as an important personal resource that helps employees in gaining back and retaining their resources. For example, those employees who are high in the psychological capital that is hope, optimism, self-efficacy, and resilience may have a “reserve” that can be utilized in stressful situations. As per the best of our knowledge, the existing literature is silent on the moderating role of PsyCap between leadership and work alienation. Having said that PsyCap can be considered as an individual resource (Hobfoll, 2001), we suggest that employees high in PsyCap avoid indulging in work alienation under an aversive leader. Hence, those employees who are high in the PsyCap are less likely to experience powerlessness, meaninglessness, and self-estrangement and ultimately better able to perform in their jobs.

## Literature Review and Hypothesis Development

### Aversive Leadership and Job Performance

With exceptions of a few studies (Pearce and Sims, 2002; Bligh et al., 2007; Thoroughgood et al., 2011), the existing literature is silent on aversive leadership. The aversive leader is defined as the one who mainly uses coercive power and gives punishments to his followers for achieving the given goals (French and Raven, 1959; Arvey and Ivancevich, 1980). Academic researchers accept the notion that aversive leaders focus more on the weaknesses of their employees and threaten them to get a performance and behavior on leader-centric goals (Yun et al., 2007). According to one study, aversive leaders yell, shout, and verbally reprimand their followers. Not only this, they may also get indulged in vulgar language (Sims et al., 2009). It is due to these negative behaviors such as intimidation and threats that employees show negative outcomes under an aversive leader (Pearce and Sims, 2002; Cox, unpublished).

Some of the negative employee and organizational outcomes associated with aversive leadership include decrease in flexibility, innovation as well as job satisfaction (Pearce et al., 2003; Podsakoff et al., 2006; Sims et al., 2009). Not only this, researchers believe that aversive leadership results in the decrease in job satisfaction and team organizational citizenship behavior (Ball et al., 1994; Yun et al., 2007). In addition to these negative outcomes, leadership researchers have a consensus on the fact that destructive leadership leads to a decrease in employee job performance (Einarsen et al., 2007; Harris et al., 2007; Schyns and Schilling, 2013; Blickle et al., 2018; Gardner and Rasmussen, 2018; Zhao, 2018). Those employees who are on the receiving side of this negative behavior are more likely to show a decrease in their performance.

As aversive leaders show unjust behavior in the form of threats and reprimands, it is not wrong to assume that employees working under an aversive leader show lower performance. This is because of the loss of resources as suggested by Hobfoll (1989) in the theory of conversation of resources. According to him, stressful situations like aversive leadership decrease employee resources. Hobfoll (1989) defined resources as “objects,” “personal characteristics,” “characteristics” or “energies.” Employees do not have enough resources, therefore, they cannot use enough resources for better job performance. Hence, we propose:

***H1: Aversive leadership is negatively related to job performance.***

### Aversive Leadership and Work Alienation

Work alienation is frequently discussed in terms of powerlessness, meaninglessness, and self-estrangement at work (Mottaz, 1981; Chiaburu et al., 2013; Shantz et al., 2015). Powerlessness refers to a feeling that the employee does not have the freedom and control in his work and that his/her actions have no impact on the organizational outcomes (Blauner, 1964). Meaninglessness is when the employee feels that his job does not contribute to the work processes. It may also be viewed as the failure to see one’s job as a part of the broader work tasks. The third dimension that is self-estrangement refers to feeling that the job lacks self-rewarding features; in other words, the only motivation left for accomplishing tasks is extrinsic needs rather than intrinsic needs (Seeman and Anderson, 1983).

Aversive leaders frequently use threats, punishment, and reprimand to get things done (Sims et al., 2009) leaving employees powerless as they have no other option than to follow their leader even if he/she is wrong. The fear of getting punished and the threat of losing a job, getting demoted, etc. make employees feel powerless as they can’t even raise their voice against the abuse of power. This results in the indulgence in work activities without any personal interest and motivation which is often called as meaninglessness and self-estrangement. An aversive leader is known for the coercive use of power (Thoroughgood et al., 2011) and it is due to this that the employees fail to utilize their skills and abilities to the fullest as a result of which they do not feel accomplished at work which is often termed as self-estrangement. The existing literature also supports the notion that aversive leadership leads to negative outcomes including but not limited to job stress, organizational deviance, job satisfaction, and team organizational citizenship behavior (Bligh et al., 2007; Saeed et al., 2017). These propositions are consistent with COR theory (Hobfoll, 2001) as well which states that stressful situation like aversive leadership results in the loss of resources such as control, freedom, and self-expression. Based on this, the current study proposes that aversive leadership leads to an increase in work alienation among employees.

***H2: Aversive leadership is positively related to work alienation.***

### Work Alienation and Job Performance

Leadership studies consider job performance as one of the major outcomes as higher job performance is the ultimate output every organization strives for (Colquitt et al., 2010). Job performance is frequently defined as the potential of employees to accomplish their respective targets, meet organizational expectations, acquire standards, or gain their organizational goals (Herman and Chiu, 2014).

Job performance has wide range application in practical research areas. It determines the success and long-term survival of an organization which is one of the several reasons researchers given an extra emphasis on understanding and exploring its antecedents (Dane and Brummel, 2014). It is revealed in many earlier studies that increasing the work involvement of an employee is a direct way to achieve better outcomes in terms of employee job performance (Anitha, 2014). Taking it the other way, those employees who are unengaged or alienated from work are less likely to show good performance. In one study done on the midwives, it was proved that work alienation is negatively related to work effort, organizational commitment, and work-life enrichment (Tummers and Den Dulk, 2013).

Employing COR theory (Hobfoll, 1989), we posit that work alienation indicates loss of resources. Feelings of powerlessness, meaninglessness, and self-estrangement together decrease the ability of employees to show better job performance. COR theory (Hobfoll, 1989) says that employees continue to strive for gaining and retaining their resources and if they feel loss of resources they avoid using conserved resources in a fear of further resource losses. As better job performance requires a lot of motivation and psychological resources to be invested, work alienation state does not allow employees to exert more due to less balance of positive resources with them. They try to conserve the available resources and avoid more consumption.

According to a study done in the manufacturing sector, work alienation not only results in emotional exhaustion, but it also decreases the well-being of employees (Shantz et al., 2014). In another study, it was found that work alienation is positively related to careerism and negatively related to career satisfaction (Chiaburu et al., 2013). Keeping in view the negative employee outcomes associated with work alienation, it is logical to propose that work alienation is negatively related with employee job performance.

According to Chisholm and Cummings (1979), meaninglessness and powerlessness (two dimensions of work alienation) have a negative relationship with the employee’s self-rated performance. Not only this, both are also responsible for management potential and progress as checked from company records. Similarly, in another study, it was found that powerlessness, meaninglessness, and self-estrangement (proxies for work alienation) have a negative relationship with self-rated extra-role performance (Suárez-Mendoza and Zoghbi-Manrique-de-Lara, 2007). In line with our theoretical stance, there are empirical evidences that work alienation leads to poor job performance (Halbesleben, 2010; Shantz et al., 2015). Hence, we extrapolate based on COR framework and the past literature that work alienation leads to a decrease in job performance.

***H3: Work alienation is negatively associated with job performance.***

### Mediating Role of Work Alienation

Work alienation has been evidenced as a negative state and related to negative work outcomes. It is believed that when employees are treated unfairly by their leaders then they start showing alienated behavior (Ensher et al., 2001). The employees showing work alienation are more likely to stay socially isolated (Nasurdin et al., 2005). Work alienation decreases work involvement as well as motivation level of employees as a result of which they start to feel a gap between their personal desires and job tasks (Banai et al., 2004; Suárez-Mendoza and Zoghbi-Manrique-de-Lara, 2007; Ceylan and Sulu, 2010). This results in a decrease in their performance as well as several other negative outcomes (Chiaburu et al., 2014; Fedi et al., 2016). In line with these evidences, the current study proposes that work alienation mediates the relationship between aversive leadership and job performance.

The COR theory also supports this relationship. According to this theory, when there is a loss of resources due to any stressful situation, people try to retain their resources as they fear if they utilize the remaining resources, they will further lose them. Working under an aversive leader is a stressful situation which leads to work alienation and poor job performance. This theory also states that individuals are continuously looking for ways to acquire and retain resources. These resources can be monetary such as money or property or non-monetary such as knowledge, time, and personal characteristics. The proposed model given in this study suggests that the employees working under an aversive leader, gone through the loss of resources due to threats and reprimands from the leader side. They get demotivated and feel no control or freedom to perform in their own way. They even perceive that their work is not meaningful to them and they avoid any engagement with their work and indulge into self-estrangement state and finally does not make efforts to perform the assigned tasks.

***H4: Work alienation mediates the relationship between aversive leadership and job performance.***

### Moderating Role of Psychological Capital (PsyCap)

Psychological capital is a well-known human capital which has received huge attention in OB and HRM literature. Luthans et al. (2004) used this term for the first time to explain the mental state of mind having four subcategories. Its first dimension “Work Self Efficacy” refers to the strong confidence in one’s ability to complete challenging tasks efficiently and effectively. The second dimension “Optimism” is a positive state of mind regarding the current and future success. The third dimension “Hope” is defined as the strong determination to successfully achieve the current goals. It also includes activities involved in changing the action plan to meet the goals successfully. The last dimension Resilience is the ability of an individual to go through the difficult situations successfully.

Psychological capital is believed to have a strong positive link with several positive employees and organizational outcomes such as job performance and work satisfaction (Luthans et al., 2007, 2010). Employees with high PsyCap are more likely to show positive behavior (Avey et al., 2010). According to a 2011 meta-analysis, PsyCap promotes positive attitudes such as job satisfaction, organizational commitment, and psychological well-being among employees and decreases negative attitudes such as cynicism, anxiety, turnover intention, and job stress. Also mentioned in the meta-analysis was the strong association between PsyCap and objective as well as subjective performance (both self-reported and peer-reported) (Avey et al., 2011).

The coping role of this important resource is evidenced from extant literature (Abbas et al., 2014). A high level of PsyCap promotes optimism at work, boost confidence and helps in achieving work goals. On the other hand, lack of this is responsible for overall dis-engagement and demotivation. Basically, it helps in nullifying the harmful stressing factors (Koller and Hicks, 2016). Based on the existing literature, the current study proposes that PsyCap moderates the relationship between aversive leadership and work alienation such that the impact of aversive leadership on work alienation will be reduced in case of high PsyCap.

The current study has taken COR theory to support the moderating role of PsyCap. This theory states that individual/dispositional factors act as resources for employees who use them to gain back and maintain their resources (Hobfoll, 1989; Hobfoll and Shirom, 2001). Those employees who work under an aversive leader face loss of resources in the form of work alienation; so, they utilize their existing resource which is PsyCap that is self-efficacy, hope, optimism, and resilience to retain and maintain their resources and stay away from negative effects of a stressful situation. We argue that people having high level of PsyCap are lesser prone to indulge in withdrawal behaviors like work alienation. PsyCap enables them to work under an aversive leader and avoid indulging in work alienation. Hence, the current study proposes the following hypothesis.

***H5: Psychological capital moderates the relationship between aversive leadership and work alienation such that the relationship will be stronger in case of low psycap and weaker in case of high psycap.***

**FIGURE 1:**
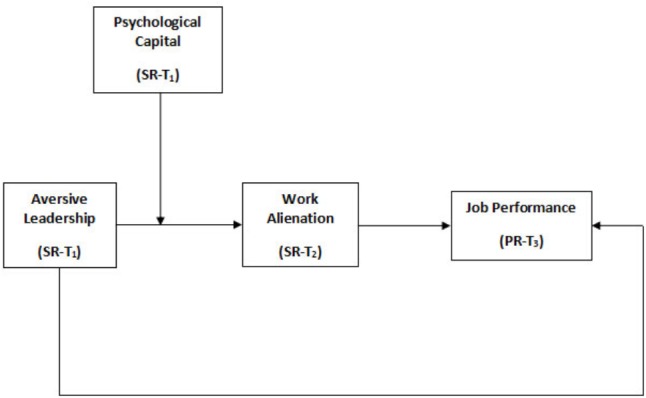
Proposed model diagram.

**Figure 1** shows the proposed theoretical framework.

## Research Design and Methodology

### Data Collection Procedure

Data were collected through questionnaire from the service sector organizations of Rawalpindi and Islamabad, Pakistan. As English is the official language of Pakistan and the majority of the people can easily read and speak English, the questionnaire was given in the English language. Past researchers did not face any language related issues while collecting the data (Naseer et al., 2016). Convenience sampling technique was used to get maximum responses. Data were collected on three time lags with an interval of 1 week each to avoid response bias. Data for aversive leadership and PsyCap was taken at time 1. Data for work alienation was collected at time 2. Performance was peer-reported and it was tapped at time 3. To avoid any nesting in peer response, we made sure that one peer responded for only one respondent.

The respondents were asked to generate a key containing first alphabets of their first and last names followed by their month of birth at time 1 with a purpose to match responses in later time responses to maintain confidentiality. They were again requested to enter the same key while filling the questionnaire given at time 2. After that, the respondents were asked to nominate any three coworkers who have been working with them for a minimum of 6 months. We, then, handed that questionnaire (which already contained the unique ID of the respondent) to his/her coworker. This made it easier for us to match the coworker response with the respondent responses. A contact person in each organization was approached by the researchers to help us to distribute and collect questionnaires from the employees who agreed to participate in the survey voluntarily. He/she also helped the co-worker to identify the main respondent, so the co-worker could rate the job performance for the main respondent more easily.

Five hundred questionnaires were distributed to employees of service sector organizations at time 1 for collecting data on an independent variable that is aversive leadership and moderating variable that is PsyCap. Four hundred thirty-four out of these 500 questionnaires were returned back. These 434 respondents were contacted after 1 week (that is at time 2) to fill the data for the time 2 variable that is work alienation. Out of these 434 respondents, only 396 respondents returned back to the questionnaire. The peers of these 396 respondents were contacted after a gap of 1 week (at time 3) to fill the questionnaire on the job performance of the respondents. Only 367 peers out of 396 submitted their responses. After removing missing values and unengaged responses, 321 questionnaires were left for final analysis.

### Sample Demographics

The demographic analysis indicated that the sample was a good representative of the population. The questions for professional experience and total experience were kept open-ended. The total experience of respondents falls between 9 months and 30 years. 72% of respondents were male while remaining was female. 64% of the total respondents had a master degree and remaining were holding bachelor degrees. 61% of employees were working as middle managers and 39% were line/front managers. 68% of the respondents were working in the private companies, 17% were working in the government organizations, whereas remaining respondents were working in the semi-government organizations.

### Measures

#### Aversive Leadership

A six-item measure was used for assessing aversive leadership. The measure is taken from (Pearce and Sims, 2002). A sample item includes “My team leader tries to influence me through threat and intimidation.” This scale has an alpha reliability value of 0.79 for the current study.

#### Work Alienation

Work alienation was measured by using an eight-item scale developed by Nair and Vohra (2009). This scale was further validated by many researchers (Nair and Vohra, 2010; Sookoo, 2013; Rollero et al., 2016). Work alienation is operationalized as a composite construct comprising of items related to its three facets, i.e., powerlessness, meaninglessness, and self-estrangement. Most of the researchers used it as an aggregate variable instead of measuring all three dimensions separately (Mottaz, 1981; Chiaburu et al., 2013; Shantz et al., 2015). We have also done the same in this study. The sample items are “I do not feel like putting my best effort at work.,” “I feel estranged/disconnected from myself.,” And “Over the years I have become disillusioned about my work.” The alpha reliability for this scale is metricconverterProductID0.84 in0.84 in this study.

#### Job Performance

A seven-item scale developed by Williams and Anderson (1991) was used for assessing the job performance of employees rated by peers. The sample item is “This person adequately completes assigned duties.” The alpha reliability value of this scale is 0.80 for the current study.

#### Psychological Capital

We used a 12-item version of the PsyCap aggregate scale developed by Luthans et al. (2006). This 12-item scale has been validated by Lorenz et al. (2016). A sample item is “When I’m in a difficult situation, I can usually find my way out of it.” In the current study, the alpha reliability value for this overall scale is 0.92.

### Data Analysis Techniques

SPSS 20 software, Process Macro by Hayes and AMOS were used for data analysis. Missing values were being treated and reverse coded items were converted into straight items before starting the analysis. Reliability analysis was done to check the consistency of the data.

**Table 1:** Results of confirmatory factor analysis (CFA).

Measurement model	χ^2^	*Df*	χ^2^/*Df*	CFI	GFI	TLI	RMR	NFI	RMSEA
**Self-reported variables**									
AL-PsyCap-WA (three factor)	**620.6**	**254**	**2.44**	**0.91**	**0.87**	**0.89**	**0.05**	**0.86**	**0.06**
AL-PsyCap-WA (one factor)	1322.5	262	5.04	0.75	0.74	0.71	0.40	0.71	0.11
**Variables at time-1**									
AL-PsyCap (two factor)	**295.79**	**120**	**2.46**	**0.94**	**0.91**	**0.92**	**0.05**	**0.90**	**0.06**
AL-PsyCap (one factor)	662.5	129	5.13	0.82	0.81	0.79	0.30	0.79	0.11
**Full measurement model**									
AL-PsyCap-WA-JP (4 factor)	**881.8**	**439**	**2.00**	**0.91**	**0.90**	**0.90**	**0.06**	**0.84**	**0.05**
AL-PsyCap-WA-JP (1 factor)	1823.5	444	4.43	0.69	0.69	0.66	0.36	0.64	0.10

*Bold values show better fit indices. AL = Aversive Leadership, PsyCap = Psychological Capital, WA = Work Alienation and JP = Job Performance.*

**Table 2:** Means, standard deviation, correlation, and Cronbach’s α reliabilities of the variables.

Sr. no	Variables	Mean	SD	1	2	3	4	5	6	7
1.	Age	28.00	12.75							
2.	Designation	–	–	0.13^∗^						
3.	Education	–	–	0.11^∗^	0.40^∗∗^					
4.	Aversive leadership	4.05	1.21	0.12^∗^	0.00	−0.09	**(0.79)**			
5.	Psychological capital	5.07	1.14	−0.04	−0.05	0.05	0.26^∗∗^	**(0.92)**		
6.	Work alienation	3.18	1.30	0.03	−0.11^∗^	−0.13^∗^	0.14^∗∗^	−0.47^∗∗^	**(0.84)**	
7.	Job performance	2.46	0.79	−0.09	0.02	0.11^∗^	−0.16^∗∗^	0.10^∗^	−0.37^∗∗^	**(0.80)**

*^∗^p < 0.05; ^∗∗^p < 0.01; ^∗∗∗^p < 0.001. N = 321; Cronbach Alpha Reliabilities are given in bold and parenthesis.*

Analysis of variance test (ANOVA) was conducted to identify those demographic variables that are not part of the study but have a significant relationship with the variables under study. The results showed that employee management level (*F* = 1.5, *p* = 0.04) and education (*F* = 2.8, *p* = 0.03) was significantly related with job performance whereas employee education (*F* = 2.1, *p* = 0.04) had a significant relationship with work alienation. These variables were controlled while conducting the analysis.

#### Confirmatory Factor Analysis

To establish the convergent and discriminant validity of measure we used confirmatory factor analysis using AMOS. Confirmatory factor analysis was done for all the variables separately to check whether the items of each variable converge significantly on the respective variable or not. The values for factor loading for all the items were above 0.3. In addition to this, a three factors CFA was also done for self-reported variables to make sure respondents understood that the three variables, namely, aversive leadership, PsyCap, and work alienation are different from each other. We also analyzed two-factor CFA for variables tapped at time 1. The results of the two factors were better than one factor. Following the recommendations of Anderson and Gerbing (1988), four-factor CFA was analyzed containing all study variables which was then compared with one combined factor CFA. The results of four-factor model showed better model fit with χ^2^ = 881.5, DF = 439, χ^2^/Df = 2.00, CFI = 0.91, NFI = 0.84, GFI = 0.90, TLI = 0.90, RMR = 0.06, and RMSEA = 0.05. Whereas the results for one-factor model were poor with χ^2^ = 1823.8, DF = 442, χ^2^/Df = 4.12, CFI = 0.69, NFI = 0.64, GFI = 0.69, TLI = 0.66, RMR = 0.36, and RMSEA = 0.10. This proved the model fitness of the proposed theoretical framework. The results are given in **Table 1**.

#### Bivariate Correlate Analysis

Mean, standard deviation, reliability, and correlation statistics are given in **Table 2**. The Cronbach’s alpha value for all the variables is greater than 0.7 (**Table 2**). The correlation statistics show a significant correlation between variables under study in expected directions. Results of the ANOVA test showed a significant impact of designation and education on the variables under study due to which these two demographic variables were controlled while conducting the analysis.

**Table 3:** Bootstrap results for direct and indirect effects.

	Path	Estimate			SE
H1	AL → JP (without mediator)	−0.22^∗∗^			0.07
H2	AL → WA	0.28^∗^			0.14
H3	WA → JP	−0.25^∗∗∗^			0.04
	AL → JP (with mediator)	−0.20^∗∗^			0.08

**Indirect effect (bias corrected confidence interval method)**					

	**Paths**	**Effect**	**SE**	**LL 95% CI**	**UL 95% CI**

H4	AL → WA → JP	−0.07	0.02	−0.06	−0.17

*N = 321. ^∗^p < 0.05; ^∗∗^p < 0.01; ^∗∗∗^p < 0.001. AL, aversive leadership; WA, work alienation, JP, job performance; SE, standard error. LLCI, lower limit confidence interval; UPCI, upper limit confidence interval. Unstandardized regression coefficients are reported. Bootstrap sample size = 2500, 95% confidence interval.*

Structural equation modeling (Chin et al., 2008) was used to test the direct and mediation hypothesis. The results of direct and indirect effect are given in **Table 3**. Structure model results indicate that the direct effect of aversive leadership on job performance (in the absence of a mediator) is significant (β = −0.22, *p* = 0.00), leading to the acceptance of hypothesis 1. The relationship between aversive leadership and work alienation (β = 0.28, *p* = 0.05) was also significant along with the relationship between work alienation and job performance (β = −0.25, *p* = 0.00) indicating that hypotheses 2 and 3 are accepted. The bootstrapping result for indirect effect between aversive leadership and job performance through work alienation is also significant (*B* = −0.07, SE = 0.02, *p* < 0.05, 95% CI: [−0.06, −0.17]). As far as weak beta coefficient value is concerned, various other studies reported the same in which the beta coefficient for indirect effect was 0.08, 0.03 (Liu and Li, 2018), and 0.08 (Qian et al., 2017) and the results were significant as well. The impact of aversive leadership remained significant on job performance even in the presence of a mediator. Sobel test was also conducted to confirm mediation results. Sobel test results (−1.90) were significant at *p* < 0.05 which confirms partial mediation leading to the acceptance of the mediation hypothesis.

**FIGURE 2:**
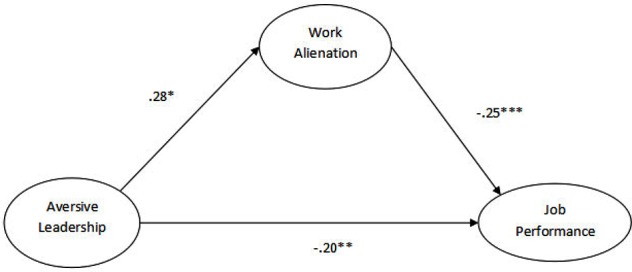
SEM path diagram.

**Figure 2** shows the SEM model with a beta coefficient and level of significance. Also given are the details for covariates. Covariates include employee designation (β = −0.24^∗∗^) and education (β = −0.15^∗^) for job performance, whereas an employee’s education (β = 0.07^∗^) for work alienation.

**Table 4:** Moderation analysis.

Moderator: psychological capital, DV: work alienation				

	**β**	**SE**	**LLCI**	**ULCI**
Constant	3.23^∗∗∗^	0.07	3.09	3.38
PsyCap	−0.20^∗∗^	0.07	−0.35	−0.05
AL	0.20^∗∗^	0.06	0.08	0.33
ALxPsyCap	−0.13^∗∗^	0.05	−0.22	−0.03
R^2^ due to interaction		0.02^∗∗^		
*F*		6.77		

**Conditional effects of moderator between aversive**				
**leadership and work alienation (slope test)**				

**Moderator: PsyCap**				
−1.14	0.35^∗∗∗^	0.09	0.17	0.53
−0.00	0.20^∗∗∗^	0.06	0.08	0.33
+1.14	0.05	0.07	−0.09	0.21

*N = 321. AL, aversive leadership; PsyCap, psychological capital; LLCI, lower limit confidence interval; ULCI, upper limit confidence interval. ^∗∗^p < 0.01, ^∗∗∗^p < 0.001.*

#### Moderation Analysis

**Table 4** shows moderation results. The current study hypothesized a moderating role of PsyCap between aversive leadership and work alienation. We used Process Macro by Haye’s to test the moderation hypothesis. The benefit of Process Macro over SPSS is that it also gives the results of slope test for high and low value of moderator, i.e., PsyCap. Interestingly, the results showed that the relationship between aversive leadership and work alienation is highly significant when PsyCap is low (β = 0.35, *p* = 0.00) but it becomes weakest and insignificant when PsyCap is high (β = 0.05, *p* > 0.05). Hence, the moderation hypothesis was also accepted.

**FIGURE 3:**
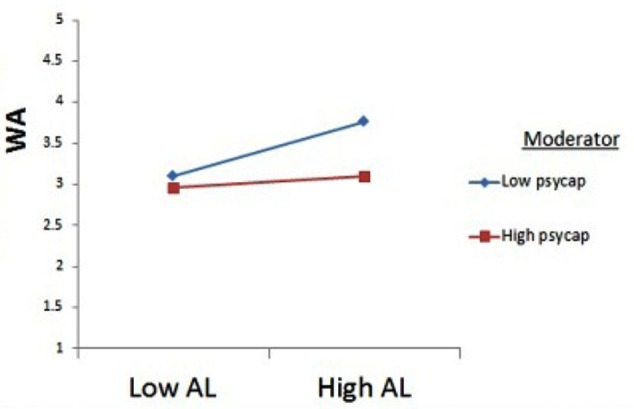
Moderating role of psychological capital between aversive leadership and work alienation.

The graphical presentation for moderation is given in **Figure 3**. It is clear from the figure that the positive relationship between aversive leadership and work alienation becomes weaker in case of high PsyCap.

## Discussion

The purpose of the current study was multifold. First, it aimed to examine the withdrawal effects among subordinates who are exposed to aversive leaders. Research on dark leadership styles has also evidenced that employees show low performance while working under this kind of leaders (Schyns and Schilling, 2013; Naseer et al., 2016; Nauman et al., 2018). But the evidence still lacks for aversive leadership-job performance relationship as a very few studies have been done on aversive leadership in particular (Pearce and Sims, 2002; Bligh et al., 2007; Thoroughgood et al., 2011).

Second, the current study filled existing gaps in the work alienation literature by identifying its antecedents and consequences. These gaps were identified by several researchers in their studies (Shantz et al., 2015; Fedi et al., 2016), but we theoretically proposed and empirically tested that subordinates get alienated from their work while working under aversive leaders. Work alienation manifest withdrawal state of employees in terms of powerlessness, meaninglessness, and self-estrangement. Although our results indicated a weak but significant indirect effect, still showing that employees get alienated from their work due to their aversive leaders and show poor performance.

Third, the study identified an important personal resource that is PsyCap which helps the employees to cope up with the damaging impact of aversive leaders and avoid getting alienated from work. The moderation results indicated that those individuals who do not have enough personal resources in the form of PsyCap experience more work alienation under an aversive leader whereas individuals with high PsyCap do not experience work alienation. This result supports our proposition that positive personal resources help to avoid resource losses.

Fourth, COR theory (Hobfoll, 1989) was employed to explain work alienation as an underlying mechanism between aversive leadership and subordinates’ performance. The COR theory (Hobfoll, 1989) explains how employees conserve, accumulate and retain resources, and avoid resource losses. We argued that unfavorable organizational conditions, such as aversive leadership, tend to drain employees’ energy levels, thereby leading them to seek to conserve their existing resources when making decisions about which activities to perform, including their performance (Hobfoll and Shirom, 2001; Hobfoll, 2001). But such behavioral responses also depend on whether employees can draw from relevant personal resources to counter the resource losses due to aversive leadership, according to COR theory (Hobfoll and Shirom, 2001; Abbas et al., 2014). It suggests that employees use their personal resources like PsyCap to gain back their lost resources. In line with COR, this study also evidenced that aversive leadership is related to the loss of resources in the form of work alienation (meaninglessness, powerlessness, and self-estrangement) among subordinates and further show poor performance. However, those employees who have more personal resources like PsyCap manage to prevent the loss of resources further. In other words, employees with high PsyCap are less likely to feel alienated from work even if they are working under an aversive leader.

### Theoretical and Practical Implications

The current study adds to the existing literature on aversive leadership by introducing the underlying mechanism through which aversive leadership affects job performance. It also validates the COR theory by examining its validity in the Pakistani context. Aversive leadership is comparatively a new concept as there is very little known about it. The current study is an effort to enhance the understanding of aversive leadership and its impact on job performance.

In addition to theoretical contributions, the current study is also useful for the practitioners. The results of this study indicate an urgent need for the business professionals to make changes in their hiring system just for making sure that people with aversive tendencies are not able to reach to the top positions. This study also suggests that employees with high PsyCap should be given jobs as they are less likely to feel work alienated under an aversive leader. Yelling, shouting, and threatening are considered normal things in Pakistani corporate sector. Leaders frequently use these tactics as a strategy thinking that the fear of punishment will increase the performance of employees but the results of this study prove that it makes the situation worse as giving threats make employees feel alienated from work which decreases their performance. The results of the current study propose that organizations should make sure that top management does not indulge in these behaviors as they may lead to work alienation and a decrease in job performance.

### Limitations and Future Research

Just like any other study, the current study has certain limitations. First, it is time-lagged study. Future researchers should conduct a longitudinal study as the nature of the association between the leader and employees change over time. Second, it only considered one outcome that is job performance. Future researchers may check the impact of aversive leadership on employee innovation, counterproductive work behavior, and turnover. Third, data was collected from only one sector. It will be fruitful to collect data from more than one sector. The current study only identified one mediator namely work alienation. Future researchers should examine the role of other mediators as well. For instance, emotional dissonance, emotional exhaustion, and job stress can be studied as mediators. Other personality traits such as Big Five, self-efficacy, and positive affectivity can also be tested as a moderator in the relationship between aversive leadership and employee outcomes. It will also be fruitful to examine the antecedents of aversive leadership. Social status, parental income, and another similar factor may play a key role in becoming a leader aversive.

## Ethics Statement

This study was carried out in accordance with the recommendations of Ethics Committee, International Islamic University, Islamabad. The protocol was approved by the Ethics Committee, International Islamic University, Islamabad. All subjects gave written informed consent in accordance with the Declaration of Helsinki.

## Author Contributions

All authors listed have made substantial contribution to the successful completion of this manuscript.

## Conflict of Interest Statement

The authors declare that the research was conducted in the absence of any commercial or financial relationships that could be construed as a potential conflict of interest.
